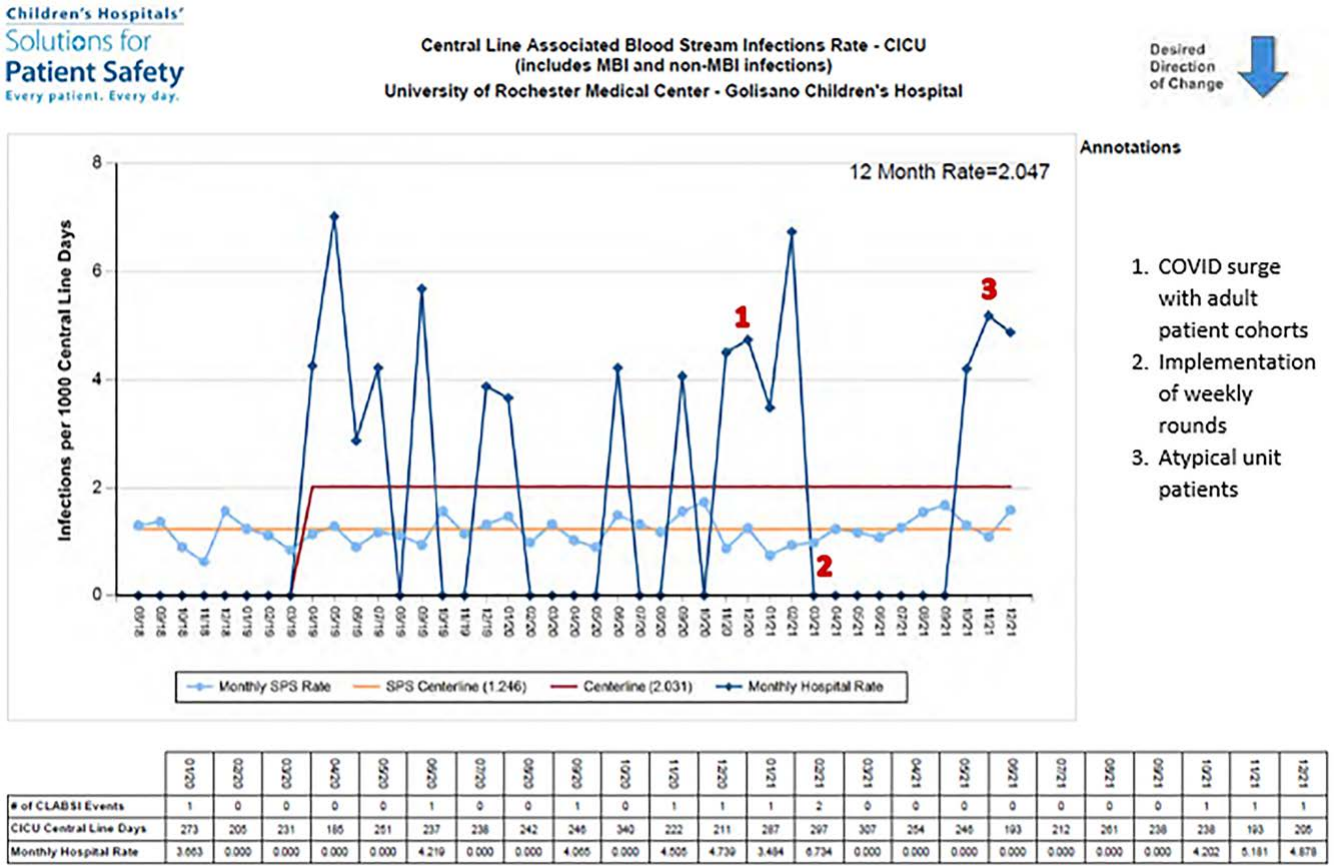# CLABSI Reduction Strategy: Utilizing Weekly Rounds with an Interdisciplinary Team

**DOI:** 10.1097/pq9.0000000000000611

**Published:** 2022-10-03

**Authors:** Amanda Welter, Jennifer Villanueva

**Affiliations:** From the University of Rochester Medical Center–Golisano Children’s Hospital Pediatric Nursing, Rochester, NY

## Introduction:

Central line-associated bloodstream infections (CLABSIs) in pediatric ICU patients lead to a wide variety of adverse outcomes, including increased mortality and morbidity, longer length of stay in the hospital, and greater healthcare costs.^1^ Pediatric cardiac ICU patients are at a significantly higher risk for these healthcare-associated infections because of an increase in risk factors, such as congenital anomalies, presence of surgical wounds, compromised immune function post cardiopulmonary bypass, and the presence of multiple invasive medical devices.^2^ After noting an increase in the pediatric cardiac ICU CLABSI rate, an interdisciplinary team was formed to approach the problem.

## Objectives:

The primary aim was to decrease the pediatric cardiac ICU’s CLABSI rate by 25% in 8 months by engaging an interdisciplinary team with identifying and addressing CLABSI maintenance bundle concerns in real time on the unit.

## Methods:

An interdisciplinary team with representatives from nursing, unit leadership, intensive care physicians, infection prevention, nurse practitioners, vascular access team, and quality improvement utilized initial Plan-Do-Study-Act (PDSA) cycles to develop a rounding tool that identified threats to the CLABSI prevention bundle with patient-specific interventions to consider implementing. Weekly interdisciplinary rounds were then conducted on all central lines. Additional PDSA cycles focused on addressing unit-specific gaps in education and increasing awareness of best practices for central line care and maintenance. These included addition and delineation of space to separate clean and dirty supplies, hygiene supplementations to the care bundle, eRecord handoff updates, and both in-person and online educational offerings.

## Results:

Preliminary data suggested a decrease in CLABSI rate during the initial 4-month intervention period (Fig. 1). The focus on central line care has also led to more consistent and robust observations for bundle compliance. In 2021, zero CLABSIs were noted for the initial 4 months after intervention contributing to a unit record of 243 days CLABSI-free. We would like to call attention to 2 points in Figure 1. Point two represents the initiation of weekly interdisciplinary CLABSI rounds. Point three represents 2 premature infants, atypical for unit census, on extracorporeal membrane oxygenation with hemodialysis catheters that required off-label use of Prismaflex.

## Conclusions:

Engagement of a microsystem’s interdisciplinary team to focus efforts on identifying and addressing real-time concerns to central lines has led to a short-term decrease in CLABSI rate. Sustaining this decrease in CLABSIs will require continual reassessment of the effectiveness of strategies utilized. Additional PDSA interventions as a result of the weekly rounds are being investigated, including the use of antimicrobial sheets for high-risk patients, the creation of unit-specific prepackaged sterile central line dressing kits, and transitioning from alcohol to chlorohexidine scrubs for hub accessing.

**Fig.**